# Comprehension of PTEN-Regulated MicroRNA Profiling in Oral Premalignant Lesions: A Critical Link to Early Detection of Oral Squamous Cell Carcinoma

**DOI:** 10.7759/cureus.82343

**Published:** 2025-04-16

**Authors:** Sanjana Gupta, Mehreen Aftab, Sandeep Sisodiya, Devi Charan Shetty, Anshi Jain, Amaresh Mishra, Showket Hussain, Nazneen Arif

**Affiliations:** 1 Department of Oral and Maxillofacial Pathology and Microbiology, ITS Center for Dental Studies and Research, Ghaziabad, IND; 2 Division of Cellular and Molecular Diagnostics (Molecular Biology), ICMR - National Institute of Cancer Prevention and Research, Department of Health Research, Ministry of Health and Family Welfare, Government of India, Noida, IND; 3 Department of Biotechnology, Parul University, Vadodara, IND

**Keywords:** gene regulation, micro-rna, oral squamous cell carcinoma, pten, tumor suppression

## Abstract

Oral premalignant lesions represent the most prevalent lesions observed within the oral cavity. These lesions generally remain asymptomatic until they advance to more severe stages, at which point their progression to oral squamous cell carcinoma (OSCC) becomes evident. Addressing this transformation necessitates further investigative efforts. The PTEN (phosphatase and tensin homolog) gene functions as a crucial tumor suppressor, with its expression regulated by a variety of complex mechanisms, including transcriptional, post-transcriptional, and post-translational processes. MicroRNAs (miRNAs) are significant modulators of PTEN expression and play an integral role in the transition from oral premalignant lesions to OSCC. This review evaluates the impact of miRNA dysregulation on PTEN and examines the continuum model of tumor suppression. It posits that the loss of PTEN functionality may transpire without alterations to the DNA sequence, particularly through mechanisms associated with miRNA regulation. Furthermore, this discourse elucidates the structural interactions between PTEN and miRNAs, particularly in the context of oral premalignant lesions, which may influence the rate of transformation to OSCC. Such insights are crucial for informing treatment strategies. The review also explores the potential of targeting specific miRNAs to restore PTEN functionality, intending to improve clinical outcomes for patients diagnosed with OSCC. By elucidating these regulatory interactions, this analysis aims to identify pathways conducive to the development of targeted therapeutic strategies.

## Introduction and background

India has emerged as a global epicenter for oral cancer, with the number of cases experiencing a significant and concerning increase daily. This alarming rise can largely be attributed to a lack of awareness and knowledge among the general population. Consequently, oral cancer presents a considerable public health challenge in the country. Lesions exhibiting dysplastic features are particularly notable due to their elevated risk of malignant transformation into oral squamous cell carcinoma (OSCC). Additionally, dysplastic lesions are commonly observed in various oral potentially malignant disorders (OPMDs). Oral premalignant lesions, such as leukoplakia, erythroplakia, lichen planus, and oral submucous fibrosis, frequently represent critical warning signs encountered by healthcare practitioners. Unfortunately, the majority of oral cancer cases in India are diagnosed at advanced stages, resulting in poor treatment outcomes and substantial financial burdens for patients and their families. This concerning prevalence highlights the urgent need for increased awareness among clinicians, improved screening methodologies, and the development of innovative treatment options [1]. The PTEN (phosphatase and tensin homolog) gene, located on the ornate landscape of chromosome 10q23.24, stands as a formidable guardian against the relentless advance of cancer. When this critical tumor suppressor is mutated or misregulated, it can unleash a cascade of malignant transformations, paving the way for various cancer forms. PTEN intricately orchestrates the phosphoinositol 3-kinase (PI3K)/protein kinase B (AKT) pathway, a vital signaling route that governs cell growth, proliferation, and programmed cell death (apoptosis) across a spectrum of tumors. As the PTEN/PI3K/AKT axis intricately influences a multitude of downstream pathways, its understanding is paramount to unraveling the complex molecular mechanisms underpinning cancer. In particular, delving into the role of microRNAs (miRNAs) as modulators of this pivotal pathway could illuminate our understanding of the transformation from benign oral premalignant lesions to the more sinister OSCC. Such insights may ultimately unveil new, innovative therapeutic strategies to combat this pervasive disease [2-4]. The "continuum model" emphasizes the significance of non-genomic factors in influencing PTEN levels, contrasting with traditional models that focus primarily on genetic mutations [5]. This framework facilitates a deeper examination of the complex relationship between PTEN regulation and miRNA interactions, with particular attention to their contributions to the evolution of oral premalignant lesions and the progression of OSCC. A comprehensive understanding of these underlying mechanisms is essential for the development of targeted therapies aimed at improving patient outcomes.

## Review

Breaking the code: decrypting the miRNA biogenesis

**Figure 1:**
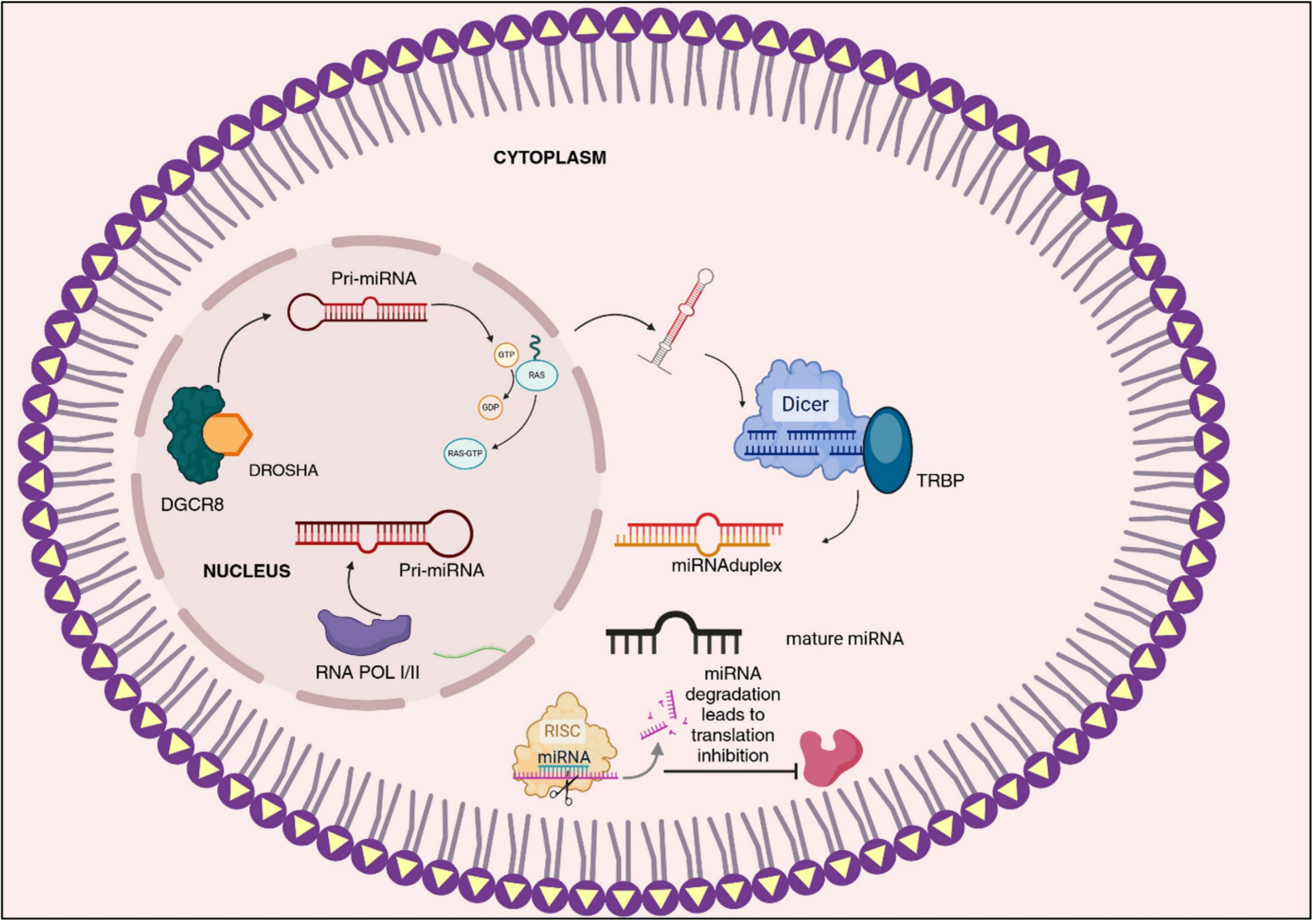
miRNA biogenesis The image was created by the author using the software "BioRender" on November 25, 2024. It illustrates miRNA biogenesis at the cellular level. MicroRNAs are small, conserved non-coding RNA molecules that regulate gene expression, transcribed by RNA polymerases II and III as precursors (pri-microRNA) before being cleaved into mature miRNAs. The biogenesis process involves two cleavage events: one in the nucleus and one in the cytoplasm. The RISC, which includes Dicer, activates to target messenger RNA indicated by the miRNA, playing a crucial role in the synthesis of various short RNAs. miRNA, microRNA; RISC, RNA-induced silencing complex

The biogenesis of miRNAs is a critical process that initiates with the transcription of specific genes into primary miRNA (pri-miRNA) transcripts. These transcripts are characterized by a 3′ polyadenylated tail, which enhances their stability, and a 5′ cap, which facilitates their processing. Following transcription, pri-miRNAs undergo a series of intricate processing steps to yield mature miRNAs, which play indispensable roles in regulating gene expression and various cellular functions [6-7]. The enzyme responsible for this transcriptional process is primarily RNA polymerase II, although RNA polymerase III also contributes by producing certain precursor microRNAs (pre-miRNAs) Figure 1 [8-9]. Central to the processing of pri-miRNAs is the microprocessor complex, comprised of the proteins Drosha and DGCR8. This complex acts on the pri-miRNA, cleaving it to form pre-miRNA structures characterized by an 85-nucleotide stem-loop configuration [10]. Once formed, these pre-miRNAs are transported out of the nucleus into the cytoplasm via the Ran/GTP/Exportin 5 complex, ensuring they reach the site of further processing. In the cytoplasm, the enzyme Dicer takes center stage, where it processes the pre-miRNAs into functional miRNA duplexes that are 20 to 22 nucleotides long [11]. These duplexes are then incorporated into the RNA-induced silencing complex (RISC), a key player in gene regulation. RISC directs the associated miRNAs to their specific target messenger RNAs (mRNAs). When miRNAs bind perfectly to their target mRNAs, they often lead to the degradation of those messages. In cases of imperfect complementarity, the interaction can inhibit protein synthesis without destroying the mRNA [12-13]. Throughout this multifaceted process, which begins with the transcription of miRNA genes and concludes with the regulated degradation of the miRNA, there are numerous control mechanisms in place to ensure proper function. However, any dysregulation in miRNA expression can have profound consequences, significantly contributing to the development of various human cancers. Such dysregulation can stem from a range of mechanisms, including chromosomal abnormalities, transcriptional changes, epigenetic modifications, and errors that occur during the synthesis of miRNAs, highlighting the importance of tightly regulating this vital cellular process [14].

MicroRNA-target interactions in modulating PTEN expression

**Figure 2:**
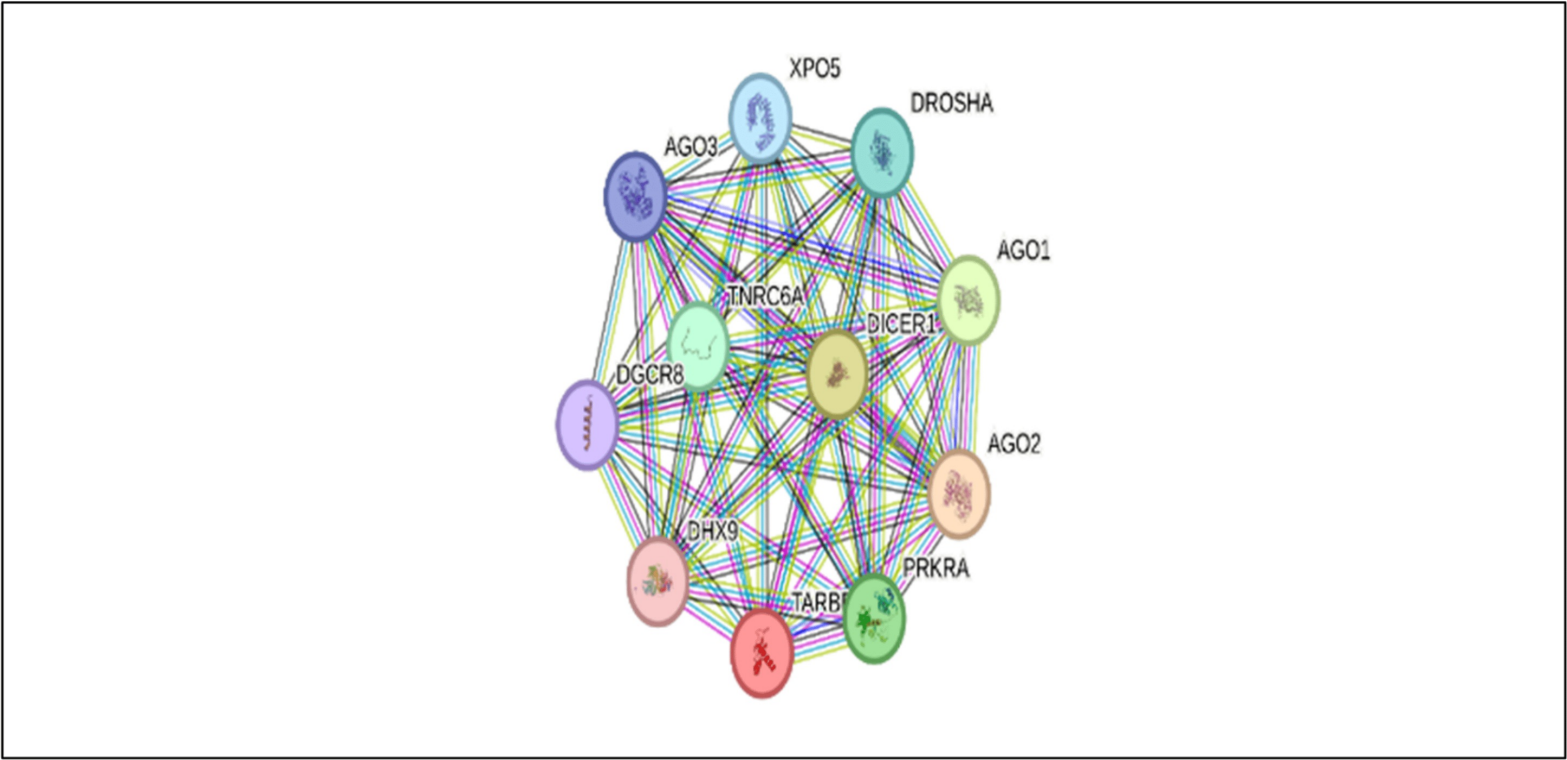
Key players in miRNA and PTEN modulation The image, extracted using the "STRING-Searched tool for the retrieval of interacting gene/Proteins" on January 30, 2025, illustrates key molecular interactions involving DROSHA, DICER1, DHX9, and DGCR8 in miRNA regulation. These miRNAs are essential for modulating the PTEN gene, a crucial tumor suppressor linked to various cancers. DROSHA and DGCR8 process pri-miRNA transcripts in the nucleus, while DICER1 generates mature miRNAs in the cytoplasm. DHX9, an RNA helicase, influences the stability and functionality of these miRNAs, affecting PTEN's post-transcriptional regulation and its signaling pathways. miRNA, microRNA

PTEN, a pivotal tumor suppressor protein, plays a vital role in regulating cell growth and division and maintaining cellular integrity. Its expression is finely tuned by various miRNAs, which are small, non-coding RNA molecules that significantly influence gene expression [15]. The reduced levels of PTEN are frequently associated with numerous types of cancer, presenting a challenge to researchers trying to establish direct causal links based solely on studies using cultured cell models [16]. One particularly noteworthy miRNA is miRNA-21, which is often found to be overexpressed in various cancers. This overabundance contributes to a decrease in PTEN levels, thereby facilitating tumor formation and progression [17]. In ovarian cancer, miRNA-124 emerges as a key player not only promoting the initiation of the disease but also contributing to the cancer cells' resistance to cisplatin, a common chemotherapy drug. Several molecules play an important role in the modulation of Pten expression by miRNAs (Figure 2). This dual role of enhancing tumor growth while enabling adaptability to treatments emphasizes the complexity of cancer biology [18]. Moreover, heightened levels of PTEN have also been implicated in a range of autoimmune disorders and lymphoproliferative diseases, illustrating the intricate relationship between this tumor suppressor and immune responses, as well as its crucial role in regulating cellular proliferation [19-20]. Various other specific miRNAs, such as miR-19a in leukemia, miR-22 in prostate cancer, and miR-26a in high-grade gliomas, have been identified as influential in modulating PTEN expression and impacting tumor dynamics, with effects varying by cancer type [21-23]. By targeting select miRNAs to boost PTEN expression, along with adjusting the activity of transcription factors that govern PTEN, researchers are exploring innovative strategies for cancer treatment. Although many newly identified miRNAs are still in the early stages of research and require further exploration, gaining a deeper understanding of their functions could pave the way for groundbreaking cancer therapies and significantly better outcomes for patients facing these challenging diseases [24].

Deciphering the molecular puzzle: PTEN and miRNA crosstalk in OPMDs and OSCCs

**Figure 3:**
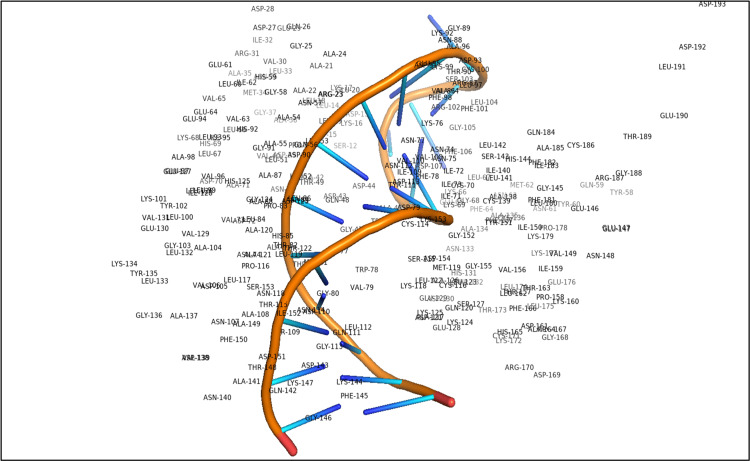
A 2D structural configuration of microRNA-derived protein and its dynamic interaction with the critical PTEN tumor suppressor gene The 2D model illustrates the complex structure of microRNA from the Protein Data Bank and its interaction with the PTEN tumor suppressor gene. This representation, created using the PyMOL visualization tool on January 30, 2025, focuses on Homo sapiens and highlights the biological significance of specific amino acids, particularly CYS 186 and ALA 98, in the binding process of microRNA to the active site of the PTEN gene. This interaction occurs in oral premalignant lesions and may lead to the progression toward oral squamous cell carcinoma. Understanding these interactions is crucial for developing targeted treatment strategies for this challenging disease.

**Figure 4:**
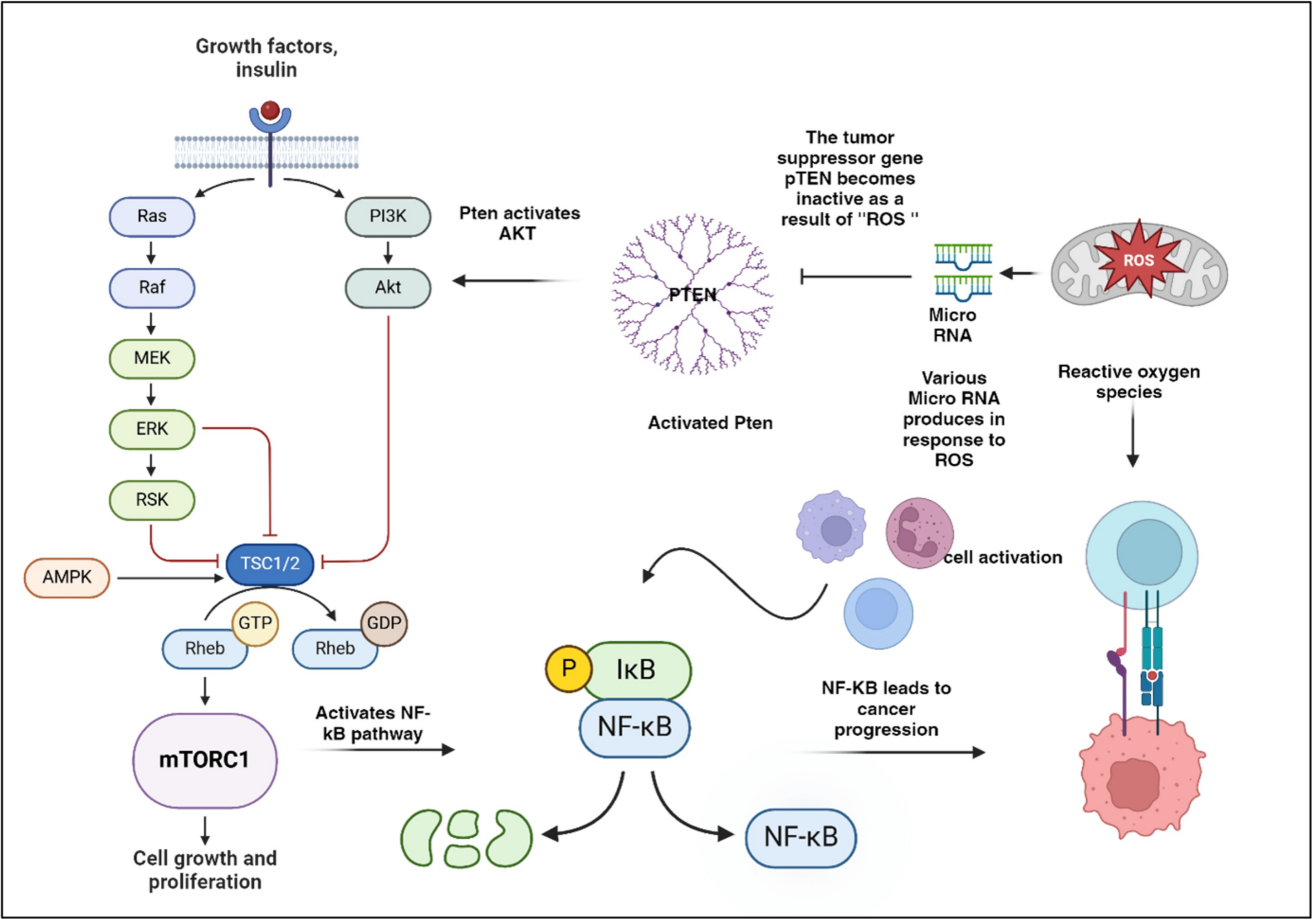
Crosstalk between PTEN and miRNA The images above are original creations by the authors using BioRender, accessed on January 1, 2025. The illustration depicts how PTEN reduces levels of PI(3, 4, 5)P3, a key second messenger in growth signaling. Beyond its role in the PI3K/AKT pathway, PTEN also acts in the nucleus, countering PI3K–AKT–mTOR signaling via its lipid phosphatase activity. It impacts various cellular processes, including survival, proliferation, metabolism, and architecture, and also regulates the NF-κB pathway, activating inflammatory responses. Additionally, mutations in PTEN lead to tumor progression through oxidative stress mediated by several miRNAs in response to ROS. miRNA, microRNA; ROS, reactive oxygen species

The pathophysiology of OSCC is linked to the dysregulation of miRNAs, which are essential for regulating cell processes such as proliferation, differentiation, and apoptosis. This dysregulation can lead to abnormal gene expression, promoting tumor development and progression. Investigating the mechanisms behind miRNA dysregulation may enhance our ability to predict patient outcomes and develop targeted therapies [25]. MiRNAs are also present in various bodily fluids, offering potential for therapeutic applications. The tumor suppressor gene PTEN plays a critical role, as different miRNAs can influence PTEN expression post-transcriptionally, affecting the amount of PTEN protein produced. This interaction is supported by an active site in the PTEN structure that facilitates its binding to the complex phosphatidylinositol 3,4,5-trisphosphate substrate along with miRNAs [26] (Figure 3). Recent insights highlight the complex interactions between miRNAs and tumor suppressor genes, which significantly impact cellular functions and cancer development. A better understanding of these interactions could lead to innovative cancer treatments [27]. Oral cancer, despite being ranked 13th in terms of mortality, poses a serious challenge to long-term survival due to factors such as recurrent lymph node metastases and high rates of local and regional recurrences [28]. These issues stem from malignant cells that evade standard treatments. Early detection of these recurrences can significantly improve survival rates, underscoring the need for highly sensitive and tissue-specific biomarkers to facilitate long-term monitoring of OSCC. Such biomarkers would help identify patients at higher risk for recurrence and support advanced restaging techniques [29]. Research has increasingly focused on the PTEN gene, a key tumor suppressor often mutated in cancers. PTEN encodes a protein that dephosphorylates important molecules, inhibiting AKT, which is involved in cell survival and proliferation. This action increases apoptosis by preventing AKT from signaling survival [30-31]. Additionally, PTEN's upregulation of p27 leads to reduced cyclin D1 levels, causing cell cycle arrest and limiting uncontrolled cell proliferation. This emphasizes the critical nature of regulatory mechanisms in cancer biology [32-33]. In addition to its pivotal role in regulating the cell cycle, PTEN serves as an essential mediator in the intricate interactions between cells and the extracellular matrix (ECM). The ECM is a sophisticated and dynamic network composed of proteins and carbohydrates that provides not only structural support to tissues but also facilitates communication between cells and their surrounding environment. PTEN's influence is critical, as it acts as a brake on several fundamental cellular processes, including cell adhesion and migration - two actions vital for tissue integrity and function [34-35]. By dephosphorylating key proteins such as focal adhesion kinase (FAK) and mitogen-activated protein kinase (MAPK), PTEN effectively modifies the signaling pathways that dictate these cellular behaviors [36-37]. This alteration allows for a significant impact on how cells interact with their surroundings, thereby shaping their responses to various external signals and maintaining their structural integrity. Consequently, PTEN plays a dynamic and essential role in the ever-changing landscape of tissue dynamics and cellular behavior [38]. Moreover, the potential for oral lichen planus (OLP) to progress to malignancy and oral dysplasia, particularly in the context of OSCC, has been intricately linked to specific miRNAs. Notably, miRNAs such as miR-4484 and miR-10b-5p have been identified as contributing factors in this transformation, while miR-200a has shown decreased expression in these cases, suggesting its possible role as a tumor suppressor [39-40]. In addition to these specific miRNAs, the miRNA miR-365 is noteworthy for its dual functions in cancer pathology; it has been shown to enhance oncogenesis and metastasis in certain cancer types while inhibiting these processes in others. Dysplasia - a condition marked by the abnormal development of tissues, including keratinocyte degeneration - stands out as a characteristic pathogenic feature of OLP, occurring alongside persistent inflammation [41]. Recent experiments utilizing an LPS-induced OLP model revealed that miR-125b specifically targets and inhibits matrix metalloproteinase-2 (MMP-2), thus blocking keratinocyte growth and promoting programmed cell death. This action occurs through the activation of the PI3K/Akt/mTOR signaling cascade, illustrating the complex interplay of signaling mechanisms involved in OLP. Furthermore, recent studies have illuminated the therapeutic potential of miRNAs, highlighting their varying levels of deregulation during the progression of oral cancer and suggesting promising avenues for future treatment strategies [42]. Research has revealed pivotal insights into the mechanisms underlying OSCC, particularly focusing on the role of specific oncogenic miRNAs (miRNAs) in cancer progression. One such miRNA, miRNA-21, is expressed at elevated levels in OSCC and is correlated with a decrease in tropomyosin and PTEN expression. This reduction in PTEN levels is particularly concerning as it plays a crucial role in regulating apoptotic processes, which, when inhibited, can foster the development and progression of cancer [43]. The underlying mechanism involves the binding of PTEN and tropomyosin (TPM1) to the 3'-untranslated region (3'-UTR) of miRNA-21, leading to an inverse relationship that disrupts normal apoptotic signaling [44]. Further exploration into the intrinsic apoptotic machinery reveals the significant regulatory role of the miR-133a and miR-133b precursors. These specific miRNAs have been shown to diminish cell proliferation in squamous cell carcinoma (SCC), suggesting that their overexpression might impede the apoptosis of cancerous cells, thereby potentially facilitating tumor progression. The relationship between miR-133a and miR-133b and their interaction with the enzyme PKM2 reinforce the complexity of these regulatory mechanisms [45]. Attention has also been directed toward RhoC and ROCK2, where studies have indicated that miRNA-138 plays a fundamental role in curtailing both the invasion and migration capabilities of Tong (SCC) cell lines. The activation of these pathways is notably tied to an increased risk of metastatic spread in OSCC, highlighting the importance of understanding miRNA interactions in cancer metastasis [46-47]. In a separate and significant investigation, the expression of miR-142-5p has been uncovered as a promoter of OSCC carcinogenesis. Various other miRNAs also target PTEN through the PI3K/AKT signaling pathway, driving alterations in cellular behavior (Table 1). Specifically, the dephosphorylation process that converts phosphatidylinositol (3,4,5)-trisphosphate (PIP3) to phosphatidylinositol (4,5)-bisphosphate (PIP2) culminates in AKT deactivation. A substantial reduction in PTEN levels disrupts normal cell growth regulation, leading to enhanced proliferation rates observed in cells with siRNA-mediated PTEN reduction. In stark contrast, cells expressing miR-142-5p mimics demonstrated marked increases in both proliferation and invasive potential, affirming the inverse relationship between PTEN downregulation and miR-142-5p overexpression, making PTEN a critical therapeutic target in OSCC [48]. Additionally, a striking finding from recent studies indicated that patients undergoing surgery for OSCC displayed markedly elevated serum levels of miR-31-5p when compared to those postoperatively. This increase suggests that miR-31-5p may serve as a reliable biomarker for differentiating oral cancer patients from healthy individuals and could play a crucial role in monitoring disease recurrence. Experimental assessments of miR-31-5p mimics and inhibitors in vitro have shown that this miRNA significantly enhances the proliferation of oral cancer cells. Further corroborating studies indicate that miR-31-5p is instrumental in facilitating tumor growth, thereby establishing a direct link with increased PTEN expression and reduced phosphorylated AKT levels. The intricate interactions within the biological landscape of OSCC point toward a promising therapeutic strategy. By specifically targeting the miRNA miR-31-5p, researchers may develop effective interventions that could inhibit tumor growth [49]. This approach takes advantage of its ability to modulate the PTEN/AKT signaling pathway, a critical pathway involved in cellular growth and survival, ultimately leading to enhanced tumor regression. Moreover, two other miRNAs, miR-142-5p and miR-21, are overexpressed in OSCC cases. These particular miRNAs interact with the PTEN gene, which normally acts as a tumor suppressor, thereby promoting cell proliferation and contributing to the aggressive nature of the disease [50-51]. In addition, variations in the gene encoding miR-196a - known as genetic polymorphisms - have been shown to significantly impact locoregional recurrence of tumors in patients. Several studies also show the intricate relationship between Pten and MTOR pathway in the pathogenesis of OSCC, which further influences the NF-KB signaling network; moreover, several miRNAs are produced in response to reactive oxygen species (ROS) as well (Figure 4). This highlights the importance of genetic factors in the behavior of OSCC. Overall, dysregulated miRNAs play a pivotal role in determining patient outcomes, affecting crucial aspects such as tumor progression, response to various therapies, rates of recurrence, metastasis to other organs, and overall survival. The influence of PTEN modulation is an important part of the OSCC cancer that makes it unique in specific form. To fully understand and leverage the potential of these molecules, more in-depth research is needed to explore the complex regulatory relationship between PTEN and the various miRNAs involved [52].

**Table 1:** Depicts various microRNAs and their function in the transformation of oral premalignant lesions to oral squamous cell carcinoma. The table above illustrates the significant roles played by various microRNAs in the intricate process of transforming oral premalignant lesions into oral squamous cell carcinoma at a cellular level. These microRNAs act as crucial regulators, influencing the molecular pathways that contribute to the progression of these lesions into a more dangerous form of cancer. Their involvement sheds light on the complex interplay of genetic factors that underpin this transformation, highlighting the subtle yet profound changes occurring within the cells that may ultimately lead to malignancy.

MicroRNA	Function
miR-10b	Not specified
miR-29b	Collagen regulation
miR-21	Not specified
miR-199- 5p	Not specified
miR-31	Cell migration and proliferation
miR-200b	Epithelial-mesenchymal transition
miR-203	Not specified
miR-200c	Not specified
miR-205	Cell differentiation and migration
miR-455- 5p	Cell differentiation and migration
miR-610	Cell adhesion and migration
miR-623	Cell migration and inflammation
miR-921	Not specified

MicroRNA translation in locoregional recurrence forecasting

**Figure 5:**
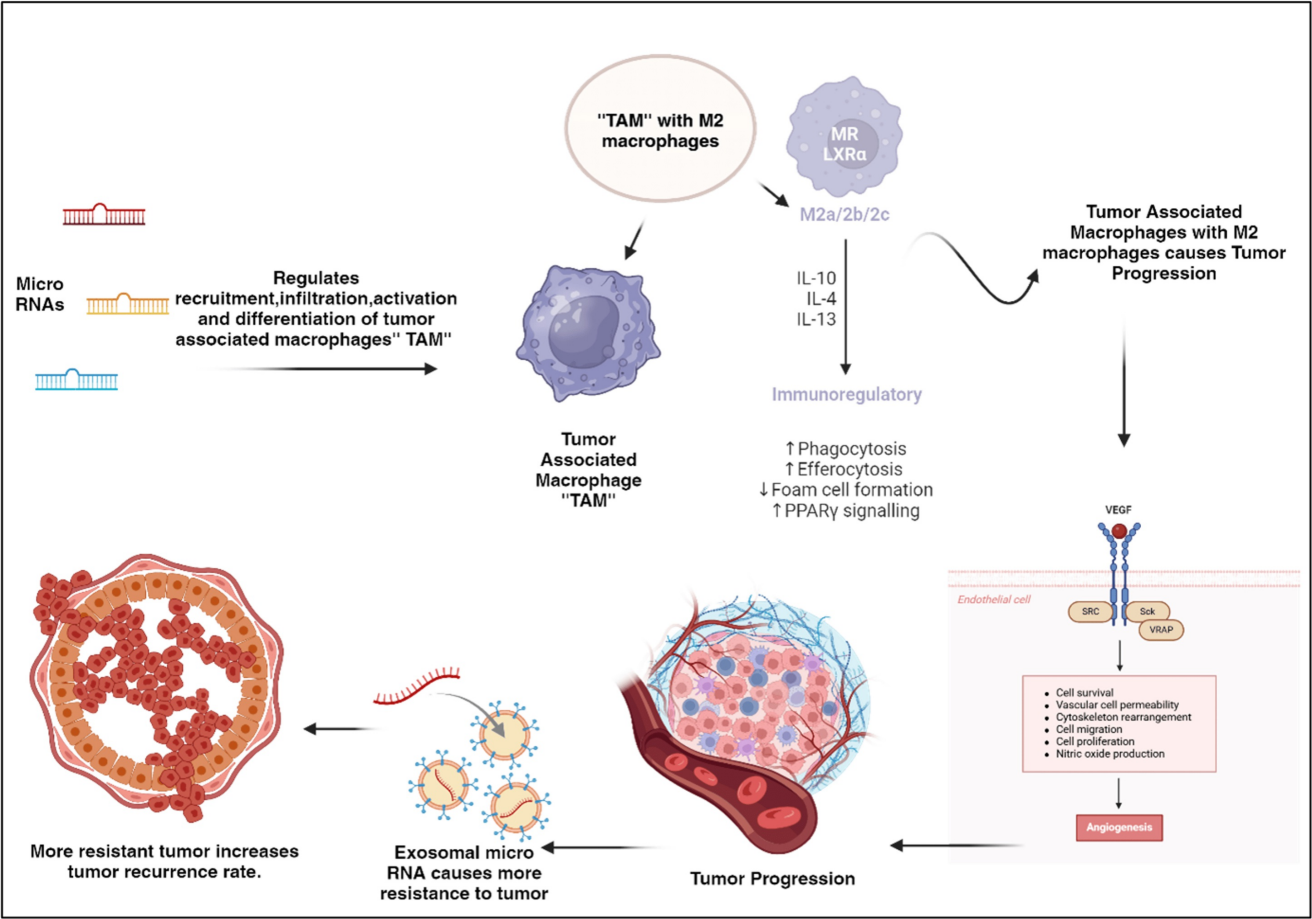
Role of miRNA in locoregional recurrence The illustration, created using BioRender on January 10, 2025, highlights the crucial roles of miRNAs in regulating genetic expression and facilitating interactions between tumor cells and TAMs within the tumor microenvironment. TAMs, prevalent in this environment, can either promote or inhibit tumor growth depending on their polarization. By influencing macrophage recruitment, infiltration, and polarization, miRNAs impact various pathways involved in tumor progression. Cytokines such as IL-10 can drive macrophage differentiation and activate growth factors such as vascular endothelial growth factor, aiding tumor advancement. Consequently, exosomal miRNAs are produced, contributing to tumor cell resistance and recurrence after treatment. miRNA, microRNA; TAM, tumor-associated macrophage

Oral malignancies present formidable challenges in treatment, necessitating a thorough and multifaceted approach. This typically involves a combination of therapeutic strategies, including surgical resection of the tumor, followed by radiotherapy and/or chemotherapy to eradicate any remaining cancer cells. For patients dealing with advanced or incurable oral cancers, the conventional treatment protocol often incorporates concurrent chemoradiotherapy with cisplatin, a powerful and widely acknowledged chemotherapeutic agent known for its efficacy against various types of cancer. However, the benefits of chemotherapy are frequently clouded by its significant toxicity, which can lead to debilitating side effects and a marked deterioration in patients' quality of life. This dilemma underscores the critical need to strike a delicate balance between achieving effective treatment outcomes and maintaining the well-being of those affected [53]. A significant obstacle in cancer treatment is the development of resistance to chemotherapy. This phenomenon can drastically undermine the effectiveness of previously successful drugs, making it a particularly concerning issue in ovarian cancer, a disease that is dauntingly associated with low survival rates. Macrophages are prevalent in the tumor microenvironment, and their polarization patterns can either support or hinder tumor development and progression. MiRNAs can control tumor growth by influencing biological characteristics such as macrophage recruitment, invasion, and polarization. Macrophage differentiation in response to cytokines, such as IL-10, IL-4, and IL-13, stimulates growth factors, particularly vascular endothelial growth factor, which promotes tumor development. As a result, exosome miRNAs are created, increasing tumor cell resistance and leading to recurrence following therapy (Figure 5). As a result, there is an urgent and pressing need for innovative, tailored treatment strategies aimed at enhancing patient outcomes and resilience against this relentless disease [54]. In recent years, researchers have increasingly focused their attention on the role of miRNAs in cancer therapy. These small, non-coding RNA molecules are integral to regulating various biological pathways that govern cancer progression and development. They hold great promise in the battle against drug resistance. By strategically modifying specific miRNAs, scientists are working to significantly bolster the effectiveness of existing therapies, potentially revolutionizing treatment protocols [55]. A comprehensive profile of miRNAs has been identified within tongue cancer, revealing intricate patterns and associations. Researchers have documented a notable upregulation of several miRNAs, including miR-214, miR-21, and miR-23a, all of which have been linked to chemotherapy resistance. Conversely, the deregulation of miR-200b and miR-15b has been observed, suggesting a complicated interplay between these miRNAs and therapeutic outcomes that requires further exploration [56-57]. Additionally, miR-98 has emerged as a pivotal player in this landscape, demonstrating a capacity to enhance resistance to the chemotherapeutic agents doxorubicin and carbapenem, particularly under hypoxic (low-oxygen) conditions. Notably, miR-98 is known to partially regulate the expression of HMGA2, a protein crucial for promoting chemosensitivity to doxorubicin, adding another layer of complexity to this dynamic [58]. A noteworthy consequence in ovarian cancer cell lines arises from the downregulation of Let-7d, a miRNA with significant regulatory functions. This downregulation triggers a cascade of events that culminates in the increased expression of proteins such as Twist and Snail. These proteins are instrumental in promoting a biological process known as epithelial-mesenchymal transition (EMT), a critical phenomenon that allows cancer cells to acquire migratory and invasive characteristics. EMT plays a fundamental role in the development of resistance against powerful chemotherapeutic agents such as 5-fluorouracil (5-FU) and cisplatin, ultimately leading to therapeutic failure and a more challenging disease management scenario. In addition to ovarian cancer, the issue of multidrug resistance (MDR) presents formidable challenges in the treatment of head and neck cancers [59]. Research has shown that altered expressions of various miRNAs are intricately linked to this phenomenon. Specifically, an increase in miR-181d, accompanied by reduced levels of miR-100 and miR-130a, creates a toxic environment that disrupts therapeutic responses to commonly used frontline treatments such as doxorubicin, paclitaxel, 5-FU, methotrexate, and cisplatin. This disruption complicates treatment strategies and poses significant hurdles for patient management. The landscape of miRNA research is continually evolving, revealing intriguing and complex correlations between specific miRNAs and their role in chemoresistance [60-61]. For instance, significant elevations in miR-155 and miR-218 have been documented in cancer cells that have developed resistance to cisplatin, opening up avenues for their potential use as biomarkers to gauge treatment efficacy. Furthermore, a striking relationship exists between cisplatin sensitivity and the expression of miR-211-5p in tongue cancer cells, a relationship that is finely regulated through the Ezrin/Fak/Src signaling pathway. Importantly, miR-211-5p displays an inverse correlation with KCNQ1OT1, underscoring its critical role in mediating chemoresistance [62]. Researchers have also made significant strides in identifying the therapeutic potential of certain miRNAs. For instance, studies indicate that the overexpression of miR-375 can reinstate sensitivity to radiation therapy by specifically targeting the IGF-1 receptor (IGF-1R). This highlights the exciting possibility of manipulating miRNA levels as a strategic therapeutic approach. By meticulously analyzing the expression patterns of miRNAs in cancer patients, researchers can unearth profound insights into the mechanisms that drive locoregional recurrence. Understanding these mechanisms is pivotal for crafting targeted therapeutic interventions tailored to the unique profiles of individual patients. Ultimately, these insights promise to lead to enhanced treatment strategies, improved clinical outcomes, and more precise prognoses [63]. By identifying and elucidating specific miRNA profiles associated with recurrence, healthcare professionals can refine risk assessments and devise personalized treatment plans. This personalized approach not only fosters more effective therapeutic outcomes but also embodies a compassionate understanding of the complexities involved in cancer care, prioritizing the well-being of patients as they navigate their treatment journeys [64].

Navigating regulatory hurdles in adopting microRNA profiling in healthcare

From a technical standpoint, miRNAs demonstrate several optimal biochemical properties that could render them highly effective indicators for various biological processes. These small RNA transcripts are notable for their remarkable stability, exhibiting a long half-life within biological specimens. This resilience means that they do not require any special handling during analysis, making them convenient for use in a wide range of available biological samples [65]. Furthermore, miRNAs can be quantified using standard laboratory techniques, such as quantitative polymerase chain reaction (qPCR), which are already widely employed in clinical settings. These methods offer advantages in terms of cost-efficiency, as they are relatively inexpensive, while also providing high levels of sensitivity and specificity [66]. While circulating miRNAs hold great promise as potential biomarkers, their integration into clinical practice faces several notable challenges. A primary obstacle is the lack of a universally accepted standard for the methodologies used to quantify miRNAs. This inconsistency can lead to significant variability in results and interpretations among different research studies, which undermines the reliability of findings [67]. Numerous research initiatives have focused on the capacity of circulating miRNAs to serve as biological markers for oral cancer, potentially leveraging their unique expression patterns to distinguish between healthy and diseased states. In the quest to identify these markers, researchers frequently utilize artificial case-control study designs. In this approach, they analyze samples from patients with various medical conditions and compare them to healthy individuals who act as controls. Although this experimental design is beneficial for molecular phenotyping and understanding disease profiles, it overlooks critical confounding factors, such as ongoing treatments and existing comorbidities. These oversights can profoundly influence the robustness and validity of the resulting conclusions. In addition to the numerous challenges already identified, the field of biomarker discovery grapples with significant issues pertaining to reproducibility, largely due to the diverse array of technologies deployed. The discrepancies in methodology can result in inconsistent outcomes, further complicating the ambitious pursuit of biomarkers that are both reliable and applicable in clinical settings [68]. Recent advancements have introduced next-generation sequencing (NGS) platforms capable of analyzing RNA samples with exceptionally low starting quantities. However, despite these innovations, many of these techniques exhibit substantial biases and lower reproducibility compared to other sequencing technologies and well-established quantitative methods such as polymerase chain reaction. This lack of reproducibility can be traced back to two main challenges. Firstly, the necessity of using limited RNA inputs often demands longer amplification cycles. This extended process can introduce minor procedural biases, which, due to the nature of amplification, become significantly exaggerated. Secondly, the resultant sequencing end products frequently reveal a preponderance of overrepresented sequences - many of which bear little to no clinical significance - thereby distorting the overall results. Despite the considerable investment of resources into miRNA research and the wealth of information already amassed, effectively translating miRNAs into long-term clinical applications hinges on collective efforts to overcome existing methodological, technical, and analytical hurdles [69]. To bolster reproducibility and credibility across various independent research endeavors, it is imperative to standardize methods for miRNA isolation and quantification. Implementing robust standard operating procedures will be crucial in this regard. Equally important is the development of comprehensive guidelines that illuminate best practices, thereby elevating the overall quality of studies conducted in this domain. Additionally, prioritizing the creation of automated and standardized assays, along with the miniaturization of current analytical methods, can facilitate the seamless transition of knowledge from the laboratory to clinical environments. Ultimately, for miRNA analysis to be successfully integrated into clinical laboratories, a thorough evaluation of its cost-effectiveness will be essential, ensuring that such innovations can be sustainably adopted and incorporated into routine diagnostic workflows [70].

## Conclusions

MicroRNAs are small, non-coding RNA molecules that play a crucial role in regulating PTEN, a tumor suppressor protein essential for the development and progression of OSCC. The dysregulation of specific miRNAs, particularly miR-21 and miR-142-5p, is closely associated with changes in PTEN expression levels. These changes can significantly influence cancer progression and patient outcomes. A comprehensive understanding of the complex signaling pathways that regulate PTEN expression is key to developing innovative therapeutic strategies aimed at enhancing PTEN activity, especially in patients with heterozygous mutations in the PTEN gene. Moreover, conditions characterized by the excessive silencing of certain miRNAs can lead to reduced PTEN levels, underscoring the urgent need for new therapeutic approaches to restore this vital protein's function. Future research should focus on identifying and characterizing the miRNAs involved in PTEN regulation, as profiling these miRNAs could be critical for the early detection of OSCC. Such advancements could facilitate the development of targeted therapies. By utilizing miRNAs as both diagnostic biomarkers and therapeutic targets, we can create significant opportunities to personalize treatment strategies, ultimately improving the management of oral cancer and providing better care for patients.
